# Comparative analysis of plasma folate, homocysteine and erythrocyte folate levels in pregnant women after folic acid administration during different pregnancies

**DOI:** 10.5937/jomb0-52621

**Published:** 2025-03-21

**Authors:** Xinglin Jin, Mei Meng, Xi Wang

**Affiliations:** 1 Huaibei Maternal and Child Health Care Hospital, Department of Obstetrics, Huaibei, China; 2 Huaibei Maternal and Child Health Care Hospital, Department of Pregnancy Care, Huaibei, China

**Keywords:** folic acid, pregnant women, homocysteine, erythrocyte folate, folna kiselina, trudnice, homocistein, eritrocitni folat

## Abstract

**Background:**

To examine and evaluate the alterations in plasma folate, homocysteine (HCY), and erythrocyte folate (FOA) concentrations among expectant mothers following folic acid supplementation in distinct pregnancies.

**Methods:**

A retrospective analysis was conducted with 416 pregnant women, divided into two groups: an observation group (n=210) who consistently took folic acid throughout pregnancy, and a control group (n=206) who only supplemented during early pregnancy. Key outcomes included plasma folate, HCY, and FOA levels, as well as pregnancy complications and neonatal outcomes.

**Results:**

Plasma folate levels were significantly higher in the observation group (10.42±2.96 ng/mL) compared to the control group (7.51±1.58 ng/mL, P<0.001). HCY levels were lower in the observation group (6.54±1.51 mmol/L) versus the control group (10.58±1.27 mmol/L, P<0.001). FOA levels were also higher in the observation group (486.42±105.29 ng/mL) compared to the control (430.20±75.12 ng/mL, P<0.001). The observation group had reduced rates of cesarean section (26.19% vs. 36.41%, P=0.025), anemia (3.81% vs. 8.74%, P=0.038), and hypertension during pregnancy (5.24% vs. 11.65%, P=0.018). Pearson correlation analysis showed a positive correlation between plasma folate and FOA (r=0.116, P<0.05) and negative correlations between plasma folate and HCY (r=-0.411, P<0.05) and between FOA and HCY (r=-0.286, P<0.05). The observation group had significantly lower FBG and P2BG levels and a reduced incidence of gestational anemia and HIP compared to the control group (P<0.05). Cesarean sections were also less frequent in the observation group. Newborns in the observation group had significantly greater height, weight, head circumference, and chest circumference than those in the control group (P<0.05).

**Conclusions:**

Consistent folic acid supplementation throughout pregnancy significantly improves maternal folate status, decreases pregnancy-related complications, and enhances neonatal health outcomes. These findings underscore the need for continuous folic acid intake during pregnancy, which could inform clinical guidelines and public health policies to optimize maternal and neonatal health.

## Abstract (sr)

**Uvod:**

Cilj je bio da se ispitaju i procene promene u koncentracijama folata u plazmi, homocisteina (HCI) i eritrocitnog folata (FOA) među trudnicama nakon dodavanja folne kiseline u različitim trudnoćama.

**Metode:**

Retrospektivna analiza je sprovedena sa 416 trudnica, podeljenih u dve grupe: posmatračka grupa (n=210) koja je dosledno uzimala folnu kiselinu tokom trudnoće i kontrolna grupa (n=206) koja je uzimala suplemente samo tokom rane trudnoće. Ključni ishodi uključivali su nivoe folata u plazmi, HCI i FOA, kao i komplikacije trudnoće i neonatalne ishode.

**Rezultati:**

Nivoi folata u plazmi bili su značajno viši u posmatranoj grupi (10,42±2,96 ng/mL) u poređenju sa kontrolnom grupom (7,51±1,58 ng/mL, P<0,001). Nivoi HCI su bili niži u posmatranoj grupi (6,54±1,51 mmol/L) u odnosu na kontrolnu grupu (10,58±1,27 mmol/L, P<0,001). Nivoi FOA su takođe bili viši u posmatranoj grupi (486,42±105,29 ng/mL) u poređenju sa kontrolnom (430,20±75,12 ng/mL, P<0,001). Grupa za posmatranje je imala smanjene stope carskog reza (26,19% naspram 36,41%, P=0,025), anemije (3,81% naspram 8,74%, P=0,038) i hipertenzije tokom trudnoće (5,24% naspram 11,65%, P= 0,018). Pirsonova korelaciona analiza je pokazala pozitivnu korelaciju između folata u plazmi i FOA (r=0,116, P<0,05) i negativnu korelaciju između folata u plazmi i HCI (r=-0,411, P<0,05) i između FOA i HCI (r=-0,286, P<0,05). Grupa za posmatranje imala je značajno niže nivoe FBG i P2BG i smanjenu incidencu gestacijske anemije i HIP u poređenju sa kontrolnom grupom (P<0,05). Carski rezovi su takođe bili ređi u posmatranoj grupi. Novorođenčad u posmatranoj grupi imala su značajno veću visinu, težinu, obim glave i grudnog koša od onih u kontrolnoj grupi (P<0,05).

**Zaključak:**

Konzistentna suplementacija folne kiseline tokom trudnoće značajno poboljšava status folata kod majke, smanjuje komplikacije vezane za trudnoću i poboljšava zdravstvene ishode novorođenčeta. Ovi nalazi naglašavaju potrebu za kontinuiranim unosom folne kiseline tokom trudnoće, što bi moglo da posluži za kliničke smernice i politike javnog zdravlja za optimizaciju zdravlja majki i novorođenčadi.

## Introduction

Folic acid, also known as vitamin M and vitamin B9, is a nutrient found in spinach. It is notable because the human body cannot produce it on its own, and must be acquired through dietary sources such as green leafy vegetables, animal liver, and bean sprouts [1]. It plays a vital role in nucleotide biosynthesis, amino acid metabolism, and numerous methylation processes, making it essential for cellular growth and reproduction. Adequate folic acid intake is particularly crucial for during pregnancy, as it is directly linked to fetal development and the prevention of birth defects, especially neural tube defects [2]. Additionally, folic acid contributes to reducing pregnancy-related complications, such as gestational diabetes and preeclampsia, by maintaining normal physiological functions [3]. However, research indicates that insufficient folic acid intake among pregnant women is a common occurrence in our country.

As pregnancy progresses, the demand for folic acid, as well as other nutrients, increases due to placental and fetal growth and development, resulting in heightened maternal folic acid metabolism. Recent research has demonstrated that the timing and dosage of folic acid supplementation can significantly influence maternal and fetal outcomes. Studies suggest that early and continuous supplementation may be more effective in optimizing maternal folate status compared to intermittent or late-stage supplementation [2]. Furthermore, the expansion of blood volume in pregnant women dilutes folic acid levels in the body, reaching its lowest point in the third trimester [4]. Hence, appropriate folic acid supplementation during pregnancy serves to promote the proliferation and growth of uterine and placental cells, foster fetal development, increase maternal blood volume, and ultimately enhance pregnancy outcomes [5] [6]. However, many pregnant women begin supplementing folic acid only after realizing its importance, often missing the critical early pregnancy stage. Moreover, factors such as dietary habits and medication use among pregnant women can impact the absorption and utilization of folic acid. Consequently, adjusting folic acid supplementation strategies according to different stages of pregnancy is crucial for improving folic acid levels in pregnant women. However, there is currently no uniform consensus on the timing of folic acid supplementation during pregnancy.

The aim of this study was to investigate the impact of folic acid administration at various stages of pregnancy on levels of plasma folic acid, homocysteine, and erythrocyte folic acid in pregnant women. The goal was to establish a scientific basis for folic acid supplementation during pregnancy. By analyzing and comparing the levels of these markers in pregnant women who received folic acid supplementation at different stages of gestation, we aimed to identify the optimal dosage and timing for folic acid supplementation, providing personalized recommendations for pregnant women. This research endeavor not only has the potential to enhance folic acid levels in pregnant women, thus preventing fetal neural tube defects and lowering the risk of birth defects, but also offers insights for developing folic acid supplementation policies in China. The findings of this study hold both practical significance and application value.

## Materials and methods

### General information

During this study, we selected a total of 416 pregnant women who received treatment at our hospital between October 2020 and October 2023. All participants met specific inclusion criteria, which required them to have a singleton pregnancy with a healthy fetus, be between the ages of 18 and 45, and have clear consciousness without any history of nervous system or mental illness. Exclusion criteria were applied to those with a previous medical history of diabetes, hypertension, or anemia, those taking medications that could impact folic acid levels, or those with a multiple pregnancy or history of abortion. The selected patients were divided into two groups based on their folic acid intake timing. The observation group included those who took folic acid before and during early pregnancy, as well as continuously during the second and third trimesters. The control group consisted of participants who only took folic acid continuously during the second and third trimesters. The study protocol was approved by the hospital ethics committee.

### Methods

The observation group supplemented folic acid 0.4 mg/d once/d for 3 months in preconception (trimester), early pregnancy (1-–13 weeks), mid-pregnancy (14–27 weeks), and late pregnancy (28–40 weeks), and took 20 days per month. The control group only supplemented folic acid in the first trimester (three months), early pregnancy (1–13 weeks), taking 20 days per month, and the dosage was the same as the observation group.

### Observation index

Laboratory parameters were assessed as follows: Venous blood samples were collected from pregnant women in the morning after fasting. The collected blood was centrifuged for 10 minutes at a speed of 3000 revolutions per minute. Careful extraction of serum was done, which was then stored at a temperature of -40°C to prevent repetitive freezing and thawing. Chemiluminescence technique was utilized to measure the level of plasma folic acid, while fluorescence polarization immunoassay was used to detect the level of plasma homocysteine (Hcy). Furthermore, 3 mL of whole blood was collected to determine hematocrit levels, and erythrocyte folic acid (FOA) levels were measured using chemiluminescence after undergoing lysis.

Evaluation of pregnancy complications focused on monitoring the incidence of pregnancy-related disorders. Incidence rates of gestational diabetes mellitus (GDM), gestational hypertension (HIP), and gestational anemia were recorded and compared between the two groups, the diagnosis time is during the prenatal examination of the entire pregnancy of the pregnant woman.

Measurement of blood glucose levels: The samples for both groups were obtained during the gestational period of 24 to 28 weeks. The levels of fasting blood glucose (FBG) and blood glucose measured 2 hours after meals (P2BG) were assessed in a state of morning fasting. P2BG was tested using a glucose tolerance test (OGTT), and patients were instructed to fasting for 8–10 hours. After fasting and venous blood collection before 8 o’clock in the morning, they were instructed to drink 75 g of glucose dissolved in 250–300 mL of warm water within 3–5 minutes. The time was counted from the first sip, and venous blood was taken and tested at 2 hours to measure blood sugar levels.

Assessment of neonatal delivery mode and nutritional status: The delivery methods utilized in both groups, including cesarean section and spontaneous delivery, were documented. Additionally, measurements of neonatal height, weight, head circumference, and chest circumference were taken and compared between the groups to evaluate the nutritional status of the newborns.

### Statistical analysis

The experimental data were analyzed using Statistic Package for Social Science (SPSS) 20.0 software (IBM, Armonk, NY, USA). The variables such as age, folic acid, and FOA were denoted as (x̄±s) in the measurement data. For comparing multiple samples, analysis of variance (F test) was employed. When the conditions for analysis of variance were not satisfied, nonparametric test (Kruskal-Wallis) was conducted for data analysis, followed by pairwise comparison using the SNK-q test. The mode of delivery, GDM, HIP, and other count data were represented as percentages (%), and the χ^2^ test was utilized. Pearson correlation was used to examine the correlation between folic acid, HCY, and FOA levels in pregnant women. The statistical results were found to be statistically significant (P < 0.05).

## Results

### Comparison of plasma parameters in two groups of pregnant women following folic acid intake

The observation group had an average age of 26.95±5.85 years and an average body mass index (BMI) of 22.63±3.15 kg/m^2^. Among them, there were 123 primiparas and 87 multiparas. In the control group, the average age and BMI were 27.01±4.33 years and 22.58±3.19 kg/m^2^, respectively, with 108 primiparas and 98 multiparas. No significant differences were observed in terms of age, BMI, and reprodu ctive history between the two groups. The observation group, which received continuous supplementation throughout pregnancy, exhibited significantly higher plasma folate (10.42±2.96 ng/mL) and FOA levels (486.42±105.29 ng/mL) compared to the control group (plasma folate: 7.51±1.58 ng/mL, FOA: 430.20±75.12 ng/mL P<0.001). In contrast, the control group showed elevated HCY levels (10.58±1.27 μmol/L) relative to the observation group (6.54±1.51 μmol/L, P<0.001). These findings suggest that continuous folic acid supplementation effectively maintains higher maternal folate levels, which are associated with lower HCY concentrations— a marker linked to various pregnancy complications. Table 1

**Table 1:** Comparison of plasma parameters in two groups of pregnant women following folic acid intake (x̄±s).

Group	N	folic acid (ng/mL)	HCY (μmol/L)	FOA (ng/mL)
Observation group	210	10.42±2.96	6.54±1.51	486.42±105.29
Control group	206	7.51±1.58	10.58±1.27	430.20±75.12
* t *		12.474	29.505	6.259
* P *		<0.001	<0.001	<0.001

### Analysis of correlation between plasma folic acid and HCY, FOA

**Figure 1:**
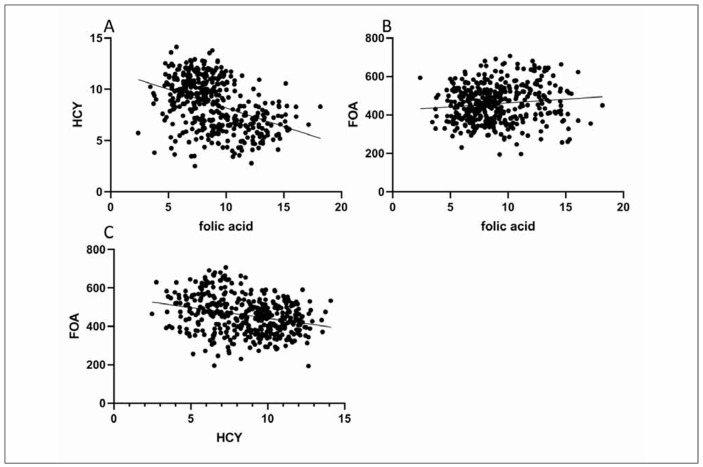
Correlation analysis of plasma folic acid with HCY and FOA. (A) correlation between plasma folic acid and HCY; (B) correlation between plasma folic acid and FOA; correlation between C:HCY and FOA.

Pearson correlation analysis revealed significant relationships between the measured parameters. A positive correlation was found between plasma folate and FOA levels (r=0.116, P=0.018), while negative correlations were observed between plasma folate and HCY (r=-0.411, P<0.001) and between FOA and HCY (r=-0.286, P<0.001). These associations suggest that higher folate levels contribute to a reduction in HCY, potentially mitigating the risk of pregnancy-related complications. Please refer to Table 2 and Figure 1 for more details.

**Table 2:** Analysis of correlation between plasma folic acid and HCY, FOA.

Index		Folic acid	HCY	FOA
Folic acid	* r *	-	-0.411	0.116
	* P *	-	<0.001	0.018
HCY	* r *	-0.411	-	-0.286
	* P *	<0.001	-	<0.001
FOA	* r *	0.116	-0.286	-
	* P *	0.018	<0.001	-

### Comparison of blood glucose levels during pregnancy between the two groups

The levels of FBG and P2BG showed a significant reduction in the observation group during pregnancy as compared to the control group, with a statistically significant difference observed (refer to Table 3).

**Table 3:** Comparison of blood glucose levels during pregnancy between the two groups.

Group	N	FBG (mmol/L)	P2BG (mmol/L)
Observation<br>group	210	4.52±1.31	7.25±1.18
Control group	206	6.50±1.63	9.05±3.05
* χ^2^*		13.641	7.910
* P *		<0.001	<0.001

### Comparison of pregnancy complications between the two groups

Table 4 highlights the incidence of pregnancy complications, including anemia, gestational diabetes mellitus (GDM), and gestational hypertension (HIP). The observation group had a significantly lower incidence of anemia (3.81% vs. 8.74%, P=0.038) and HIP (5.24% vs. 11.65%, P=0.018) compared to the control group. These differences indicates a notable decrease in the occurrence of anemia and HIP during pregnancy in the observation group compared to the control group.

**Table 4:** Comparison of pregnancy complications between the two groups (N, %).

Group	N	Anemia of<br>pregnancy	GDM	HIP
Observation<br>group	210	8 (3.81)	13 (6.19)	11 (5.24)
Control group	206	18 (8.74)	20 (9.71)	24 (11.65)
* χ^2^*		4.311	1.763	5.549
* P *		0.038	0.184	0.018

### Comparison of nutritional status and delivery mode between the two groups

The observation group exhibited better neonatal outcomes, with newborns showing significantly higher measurements in height, weight, head circumference, and chest circumference compared to those in the control group (P<0.05). Additionally, the rate of cesarean sections was significantly lower in the observation group (26.19% vs. 36.41%, P=0.025). These findings suggest that consistent folic acid supplementation enhances fetal development and supports normal delivery, reducing the likelihood of cesarean sections. This improvement in neonatal outcomes is likely due to the continuous availability of folate, which supports optimal fetal growth and reduces complications that may necessitate surgical delivery. Please refer to Table 5 for detailed information.

**Table 5:** Comparison of nutritional status and delivery mode between the two groups (N, %).

Index	Time	Observation group (n=210)	Control group (n=206)	* t/χ^2^*	* P *
delivery mode	cesarean section	55 (26.19)	75 (36.41)	5.053	0.025
	normal childbirth	155 (73.81)	131 (63.59)		
Height (cm)		51.86±1.85	50.71±2.39	5.494	<0.001
Weight (g)		3512.46±415.08	3426.85±457.10	2.001	0.046
Head circumference (cm)		35.52±1.38	34.67±1.08	6.987	<0.001
Chest size (cm)		34.52±4.02	33.64±4.11	2.208	0.028

## Discussion

Pregnancy represents a crucial phase in the growth and development of the fetus, and maintaining an appropriate dietary structure can significantly contribute to the fetus’s overall development. Folic acid, an essential component of biological protein, pantothenic acid, and nucleotide synthesis, cannot be produced within the body. Unfortunately, the folic acid content found in regular diets falls short of meeting the needs of both the fetus and the mother during pregnancy. The level of folic acid has a profound impact on fetal growth and development. Firstly, folic acid plays a pivotal role in the development of the fetal neural tube, making it a critical nutrient. If there is a deficiency in folic acid, neural tube defects such as cerebrospinal canal insufficiency may occur. Moreover, folic acid is directly involved in DNA synthesis and plays a significant role in cell division, as well as the development of fetal organs. Research indicates that inadequate folic acid levels in pregnant women restrict fetal development and increase the likelihood of complications such as premature delivery and low birth weight [7] [8]. Consequently, proper folic acid supplementation is crucial in promoting fetal growth and development, as well as enhancing maternal blood volume. Nevertheless, the timing of folic acid supplementation remains a topic of controversy, and determining the optimal time to supplement folic acid has become a central concern for obstetricians and nurses.

Currently, the primary method for assessing folic acid content is by measuring plasma folic acid levels. However, variations in plasma folic acid metabolism and short-term folic acid intake can potentially interfere with plasma folic acid levels [9] [10]. Con sequently, folic acid can reflect the metabolic levels of folic acid in humans. In cases of folic acid deficiency, the folic acid level of humans is first detected by folic acid (FOA) levels, which accurately represent the storage of folic acid in the body [11] [12]. In the present study, the control group exhibited significantly lower levels of plasma folic acid and FOA, while the level of HCY was significantly higher compared to the observation group. These findings indicate that folic acid supplementation during pregnancy effectively increases folic acid content in pregnant women while reducing HCY levels, with continuous supplementation showing better results in the second and third trimesters. This is because folic acid deficiency is less pronounced in early pregnancy, and the demand for folic acid further increases in the latter stages, intensifying the degree of folic acid deficiency. Early-stage folic acid supplementation helps maintain high folic acid levels in the body, thereby meeting the increased demand for folic acid during the second and third trimesters of pregnancy and preventing rapid declines in folic acid and FOA levels before delivery [13].

Not only does folic acid play a role in normal fetal growth and development, but it also functions to prevent the occurrence of pregnancy complications [14]. This study found that the observation group had a significantly lower incidence of pregnancy anemia and HIP compared to the control group, suggesting that early supplementation of folic acid may contribute to reducing the occurrence of pregnancy complications. This can be attributed to the fact that insufficient levels of folic acid indicate inadequate maternal nutrition supply. Insufficient maternal nutrition can increase the likelihood of experiencing anemia during pregnancy [15]. Additionally, HCY is caused by metabolic issues with methionine and cysteine. After methionine supplies a methyl group, it is metabolized into HCY. In order for HCY to be converted back into methionine, it needs to accept a methyl group transferred by N5-methyltetrahydrofolic acid. However, if the level of folic acid is too low, the production of N5-methyltetrahydrofolic acid can be hindered, preventing the conversion of HCY into methionine. Consequently, this leads to an elevation in HCY levels in the body. HCY can cause complications such as HIP and GOM through endothelial cell injury, hypovascularization of chorionic villi, and abnormal embryonic development. Pearson correlation analysis in this study revealed a significant positive correlation between plasma folic acid and FOA, a significant negative correlation between plasma folic acid and HCY, and a significant negative correlation between plasma FOA and HCY. This further demonstrates the mutual interference and interaction among folic acid, FOA, and HCY. Regular monitoring of their levels facilitates a dynamic evaluation of maternal pregnancy, allowing for adjustments in folic acid supplementation and diet, and ultimately supporting women in achieving a healthy delivery. The early and third trimesters of pregnancy play a crucial role in the physical growth and development of the fetus. In this study, the incidence of cesarean section was significantly lower in the observation group compared to the control group. Additionally, newborns in the observation group exhibited significantly higher measurements in terms of height, weight, head circumference, and chest circumference compared to those in the control group. These findings indicate that the continuous administration of folic acid during the second and third trimesters can effectively enhance the nutritional status of newborns, decrease the likelihood of cesarean section, and improve maternal and fetal outcomes. This may be attributed to the fact that insufficient folic acid intake during pregnancy can disrupt iron metabolism, leading to iron deficiency anemia [16]. Anemia in pregnant women not only weakens the uterus but also results in inadequate placental perfusion and oxygen supply. This, in turn, can lead to fetal distress, neonatal asphyxia, and hypoxicischemic encephalopathy. Moreover, anemia can compromise the production of antibodies in pregnant women, weaken immune function, elevate the risk of infection, and increase the chances of premature delivery and low birth weight. Furthermore, insufficient folic acid intake can contribute to gestational hypertension. Patients with hypertensive disorder complicating pregnancy often experience varying degrees of hemorrhagic spasm, leading to inadequate blood supply to the placenta, degeneration of villi, diminished placental function, intrauterine hypoxia, neonatal asphyxia, and fetal distress [17].

Despite the valuable insights provided by this study, there are several limitations that should be acknowledged. First, the retrospective design limits the ability to establish causality between folic acid supplementation and the observed outcomes. Second, the study was conducted at a single medical institution, which may limit the generalizability of the findings to broader populations. Third, although we controlled for certain variables, other potential confounding factors, such as dietary habits and genetic variations in folate metabolism, were not accounted for in this study. Additionally, this study did not differentiate between types or doses of folic acid supplements, which could provide more nuanced insights into optimal supplementation strategies. To address these limitations, future studies should consider a prospective, multicenter design to enhance the generalizability of the findings. Investigating the influence of genetic factors could provide more personalized recommendations for folic acid supplementation. Moreover, randomized controlled trials comparing different doses and forms of folic acid (e.g., folic acid versus 5-methyltetrahydrofolate) throughout pregnancy would offer clearer guidance on optimizing supplementation regimens. Long-term follow-up studies could also assess the impact of maternal folic acid supplementation on children’s developmental and health outcomes, providing valuable data on the long-term benefits of folate optimization during pregnancy. In conclusion, the administration of folic acid prior to and during the early stages of pregnancy, along with continuous supplementation during the second and third trimesters, can effectively enhance folic acid and FOA levels in expectant mothers. This regimen facilitates the optimal growth and development of the fetus, enhances the nutritional status of newborns, and reduces the likelihood of cesarean section. Additionally, early folic acid supplementation contributes to a decreased occurrence of pregnancy-related complications, ultimately improving the overall prognosis for both mothers and infants.

Based on the findings of this study, it is recommended that China enhance policy advocacy, public awareness, and education regarding the utilization of folic acid during pregnancy in order to enhance the awareness among pregnant women about the significance of folic acid. The government can implement the following strategies: firstly, establish guidelines for the administration of folic acid during pregnancy, specifying the appropriate dosage, timing, and precautions to serve as a reference for pregnant women. Secondly, provide comprehensive training and guidance to medical institutions to enhance the expertise of healthcare professionals in prescribing folic acid during pregnancy. Thirdly, conduct extensive awareness campaigns through diverse channels to remind pregnant women about the importance of folic acid supplementation during pregnancy and to reduce the risk of conditions such as fetal neural tube defects. Additionally, it is encouraged for research institutions to conduct further scientific studies on folic acid to furnish a more robust evidence base for its use during pregnancy.

## Dodatak

### Funding

This work was supported by the Research project of Anhui Provincial Health Commission in 2022 »Correlation study on the effects of folic acid supplementation at different stages of pregnancy on GDM and blood glucose levels« [Project number: AHWJ2022c047].

### Conflict of interest statement

All the authors declare that they have no conflict of interest in this work.
